# Mouse strain-dependent variation in metabolic associated fatty liver disease (MAFLD): a comprehensive resource tool for pre-clinical studies

**DOI:** 10.1038/s41598-023-32037-1

**Published:** 2023-03-22

**Authors:** Hamzeh Karimkhanloo, Stacey N. Keenan, Jacqueline Bayliss, William De Nardo, Paula M. Miotto, Camille J. Devereux, Shuai Nie, Nicholas A. Williamson, Andrew Ryan, Matthew J. Watt, Magdalene K. Montgomery

**Affiliations:** 1 Department of Anatomy and Physiology, School of Biomedical Sciences, Faculty of Medicine Dentistry and Health Sciences, University of Melbourne, Melbourne, VIC 3010 Australia; 2 Metabolism, Diabetes and Obesity Program, Monash Biomedicine Discovery Institute, and Department of Physiology, Monash University, Clayton, VIC Australia; 3 Melbourne Mass Spectrometry and Proteomics Facility, Bio21 Molecular Science and Biotechnology Institute, The University of Melbourne, Melbourne, VIC Australia; 4 TissuPath, Mount Waverley, VIC 3149 Australia

**Keywords:** Metabolic disorders, Nutrition disorders

## Abstract

Non-alcoholic steatohepatitis (NASH), characterized as the joint presence of steatosis, hepatocellular ballooning and lobular inflammation, and liver fibrosis are strong contributors to liver-related and overall mortality. Despite the high global prevalence of NASH and the substantial healthcare burden, there are currently no FDA-approved therapies for preventing or reversing NASH and/or liver fibrosis. Importantly, despite nearly 200 pharmacotherapies in different phases of pre-clinical and clinical assessment, most therapeutic approaches that succeed from pre-clinical rodent models to the clinical stage fail in subsequent Phase I-III trials. In this respect, one major weakness is the lack of adequate mouse models of NASH that also show metabolic comorbidities commonly observed in NASH patients, including obesity, type 2 diabetes and dyslipidaemia. This study provides an in-depth comparison of NASH pathology and deep metabolic profiling in eight common inbred mouse strains (A/J, BALB/c, C3H/HeJ, C57BL/6J, CBA/CaH, DBA/2J, FVB/N and NOD/ShiLtJ) fed a western-style diet enriched in fat, sucrose, fructose and cholesterol for eight months. Combined analysis of histopathology and hepatic lipid metabolism, as well as measures of obesity, glycaemic control and insulin sensitivity, dyslipidaemia, adipose tissue lipolysis, systemic inflammation and whole-body energy metabolism points to the FVB/N mouse strain as the most adequate diet-induced mouse model for the recapitulation of metabolic (dysfunction) associated fatty liver disease (MAFLD) and NASH. With efforts in the pharmaceutical industry now focussed on developing multi-faceted therapies; that is, therapies that improve NASH and/or liver fibrosis, and concomitantly treat other metabolic comorbidities, this mouse model is ideally suited for such pre-clinical use.

## Introduction

Non-alcoholic fatty liver disease (NAFLD) is the most common chronic liver condition in developed countries, with a global prevalence of 24%^1^. Non-alcoholic steatohepatitis (NASH) is a severe form of NAFLD, which is characterized as the presence of steatosis (liver lipid exceeding 5% of liver weight), hepatocellular ballooning and lobular inflammation, in the absence or presence of various degrees of fibrosis^1,2^. NASH and liver fibrosis can further proceed to end-stage liver diseases, such as cirrhosis and hepatocellular carcinoma (HCC)^3,4^, and liver fibrosis is a strong contributor to liver-related and overall mortality^5^. NASH is associated with a variety of metabolic co-morbidities, including obesity (82% prevalence), type 2 diabetes (T2D, 48%) and dyslipidaemia (82%)^6–8^. These associations have prompted some to view NASH as the hepatic manifestation of advanced metabolic disease, leading to a redefinition of this disease cluster to metabolic (dysfunction) associated fatty liver disease (MAFLD)^9^.

Despite the substantial global burden on healthcare systems, there are currently no FDA-approved therapeutic strategies for preventing or reversing NASH and/or liver fibrosis^10^. With nearly 200 pharmacotherapies being in different phases of pre-clinical and clinical trials, to date, only a few have showed the anticipated improvements in NASH and fibrosis (i.e., obeticholic acid, elafibranor, selonsertib, cenicriviroc, and resmetirom)^11^. Importantly, most therapeutic targets that prove promising in pre-clinical mouse models do not show the same desired effects in humans. This is not surprising considering the current lack of adequate mouse models that fully recapitulate the many aspects of the human disease. Ideally, such a mouse model should be diet-induced to mimic chronic human overnutrition, show all features of histologically defined NASH and liver fibrosis, and display characteristic metabolic comorbidities, including obesity, hyperglycemia, glucose intolerance, insulin resistance, dyslipidaemia, and cardiovascular disease^6^.

Inbred mice are the most popular animals used for liver research due to their availability and low cost, with the C57BL/6 strain being the workhorse in these studies^12^. While NASH can be induced through different experimental approaches, each design has its limitations. For example, while feeding mice a methionine choline deficient (MCD) diet leads to NASH with severe fibrosis and *bona fide* ballooning degeneration, the mice lose rather than gain weight due to hepatotoxicity^12^. The MCD diet as well as carbon tetrachloride (CCl_4_) dosing, a hepatotoxin that leads to rapid liver fibrosis, are not representative of human disease progression, with CCl_4_ leading to genotoxicity and oxidative DNA damage^13^. In addition, genetic models of obesity/diabetes (e.g., *ob/ob*) or NASH (e.g., PTEN^-/-^, PPARα^-/-^ mice) either do not develop NASH or do not show all characteristic metabolic comorbidities^13^. Typical western diets consumed by humans contain high levels of lipids, sucrose, fructose and cholesterol. However, while mice fed such western-style diets develop obesity, insulin resistance and hepatic steatosis, several studies have shown that features of NASH are not pronounced in this dietary model^14^, with the degree of steatosis, inflammation and fibrosis impacted by dietary composition, treatment duration, and most importantly, the rodent strain^15^. Given the vast differences in susceptibility of inbred mouse strains to hepatic steatosis^16,17^, NASH and liver fibrosis^18^, this suggest that the genetic background and dietary exposure might be important for NASH induction, however, this remains unexplored in a systematic manner.

Given the lack of mouse models that adequately recapitulate NASH pathology in the presence of NASH-associated metabolic comorbidities, particularly utilizing a western diet to mimic chronic human overnutrition, this study aimed to provide an in-depth comparison of NASH pathology and markers of the metabolic syndrome in eight commonly available inbred mouse strains, offering a comprehensive resource tool for future pre-clinical studies in liver disease.

## Results

### Mouse strains show substantial variation in NASH pathology and liver fibrosis

**Figure 1:**
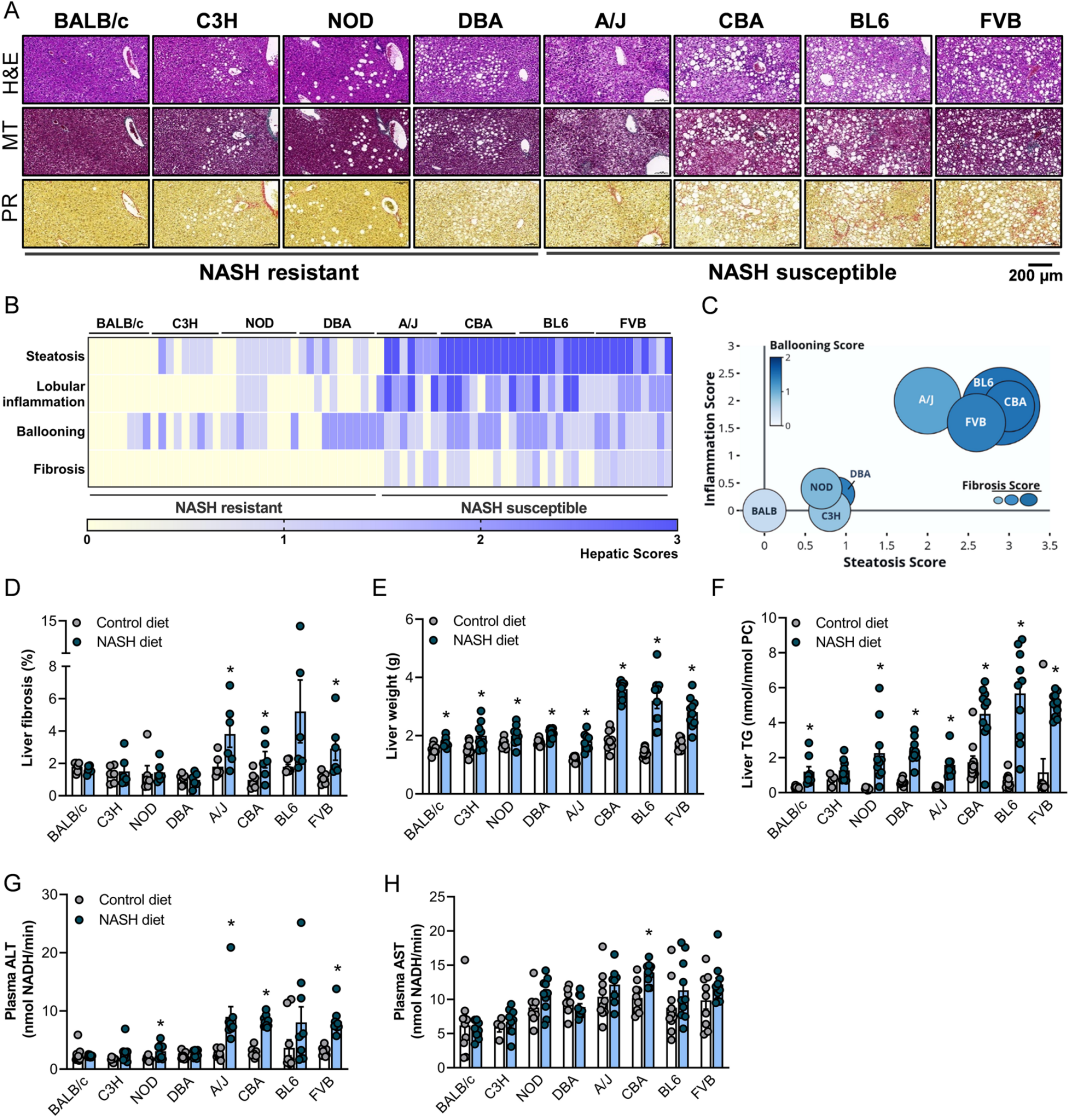
Liver histopathology assessment identifies NASH sensitive and NASH resistant mouse strains. (**A)** Representative liver histology (H&E, Masson’s Trichrome and Picrosirius Red staining) in A/J, BALB/c, C3H/HeJ, C57BL/6J, CBA/CaH, DBA/2J, FVB/N and NOD/ShiLtJ mice fed a western-style diet, sorted by increasing prevalence of NASH and liver fibrosis. (**B**) Heat map analysis showing hepatic scores for steatosis, lobular inflammation, hepatocyte ballooning and fibrosis, (**C**) comparison of hepatic steatosis, inflammation, hepatocyte ballooning and fibrosis across all eight mouse strains, (**D**) percentage hepatic fibrosis (n = 6/group), (**E**) liver weight (n = 8–11/group), (**F**) liver triglyceride content (n = 7–10/group, n = 4 C3H Chow), (**G**) plasma alanine aminotransferase (ALT) (n = 8–10/group) and (**H**) plasma aspartate aminotransferase (AST) (n = 8–10/group, n = 4 C3H Chow) activity, all sorted by increasing prevalence of NASH and liver fibrosis (as assessed by histopathological grading). Data are means ± SEM, *p < 0.05 vs. respective controls, as assessed by two-way unpaired t-test.

All eight mouse strains were fed the NASH or Control diet for a maximum of 32 weeks. The prevalence of NASH and liver fibrosis varied substantially across mouse strains, as shown by representative histopathological H&E, Masson’s Trichrome and Picrosirius Red staining (Fig. 1A). Mouse strains were classified as ‘NASH resistant’ or ‘NASH susceptible’ based on hepatic scores for steatosis, lobular inflammation, hepatocyte ballooning and fibrosis (Fig. 1B). Representative histopathology for the Control mice is shown in Fig. S1. BALB/c mice were completely resistant to NASH and liver fibrosis, with complete absence of hepatic steatosis and inflammation (Fig. 1A,B), while C3H, NOD and DBA mice only showed mild steatosis, in the absence or presence of low grades of lobular inflammation and hepatocyte ballooning. In contrast, A/J, CBA, BL6 and FVB/N mice (Fig. 1A,B) were susceptible to NASH and liver fibrosis, with co-presence of substantial steatosis (grades 2–3), lobular inflammation (grades 2–3), hepatocyte ballooning (grade 1–2) and modest fibrosis (grade 1a) (Fig. 1C, see Table S3 for the respective NAS scores). NASH-related fibrosis was confirmed in A/J, CBA, BL6 (p = 0.11) and FVB/N mice using quantitative Orbit Image Analysis (Fig. 1D).

As expected, the livers of all mice fed the NASH diets were heavier compared with the respective Control livers, with the most pronounced increase in liver weight observed in CBA, BL6 and FVB/N mice (1.6–2.3-fold increase; Fig. 1E). Similarly, liver triglyceride content was increased in all eight mouse strains (C3H p = 0.14), with the greatest 2.6–8.6-fold increase again observed in CBA, BL6 and FVB/N mice (Fig. 1F). Plasma alanine aminotransferase (ALT) is commonly assessed as a marker of liver injury. ALT levels were increased in the four mouse strains (A/J, BL6 p = 0.15, CBA and FVB/N) that exhibited histopathologically confirmed NASH and liver fibrosis, as well as in NOD mice, but not in the other three NASH-resistant strains (Fig. 1G). In contrast, plasma aspartate aminotransferase (AST) was only elevated in CBA mice fed the NASH diet (Fig. 1H).

### Gene expression analysis corroborates NASH histopathology

**Figure 2:**
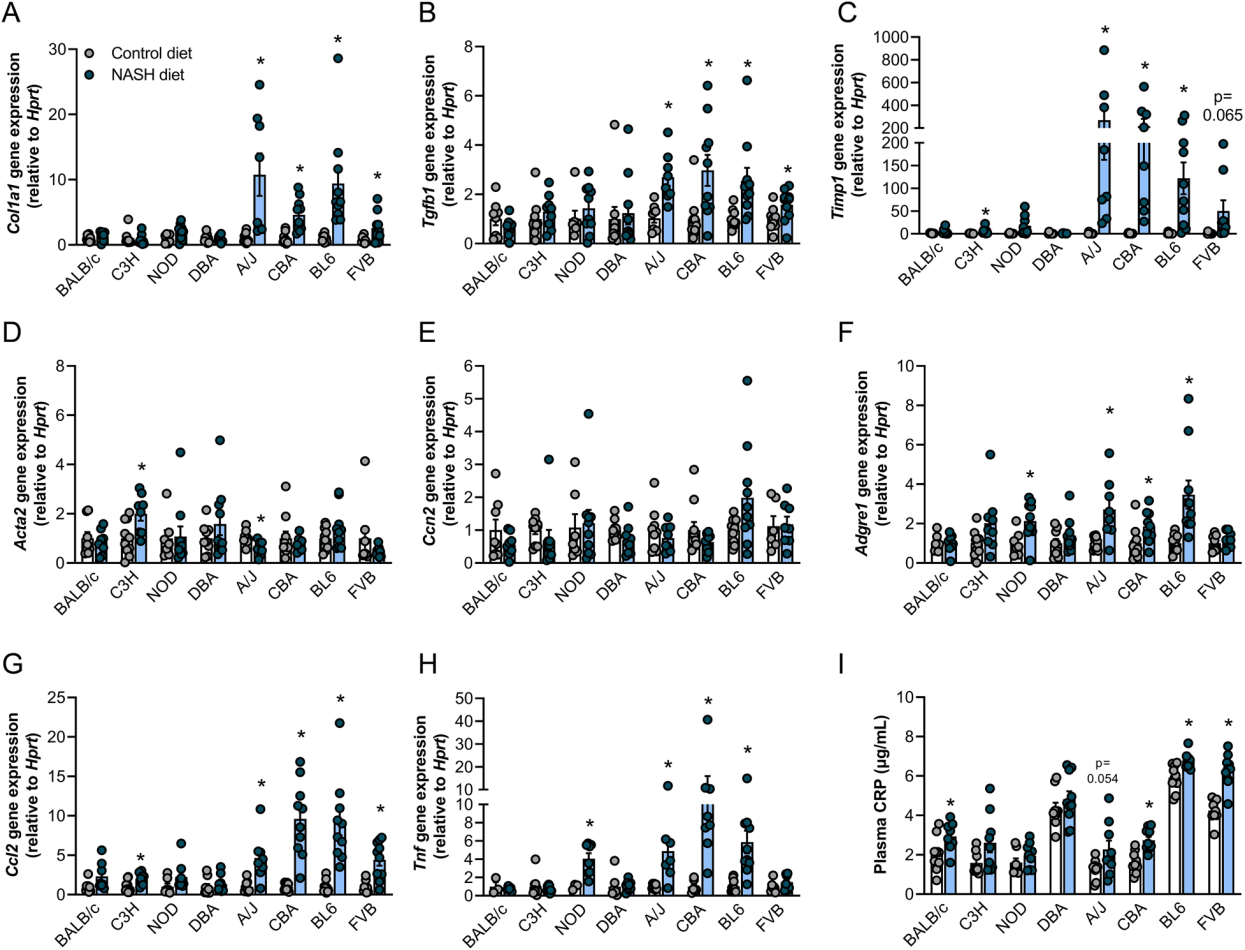
Analysis of genes encoding profibrotic and proinflammatory proteins in the liver. mRNA expression was assessed in the livers of Control (brown) and western diet-fed (blue) A/J, BALB/c, C3H/HeJ, C57BL/6 J, CBA/CaH, DBA/2 J, FVB/N and NOD/ShiLtJ mice, including (**A**) α-1 type I collagen (*Col1a1*) (n = 8–10/group), (**B**) transforming-growth factor-1 (*Tgfb1*) (n = 7–10/group), (**C**) tissue inhibitor of metalloproteinases 1 (*Timp1*) (n = 6–10/group), (**D**) α-2 actin (*Acta2*) (n = 8–10/group), (**E**) connective tissue growth factor (*Ccn2/Ctgf*) (n = 6–10/group), (**F**) adhesion G protein-coupled receptor E1 (*Adgre1 / F4/80*) (n = 8–10/group), (**G**) chemokine (C–C motif) ligand 2 (*Ccl2 / Mcp1*) (n = 7–10/group) and (**H**) tumour necrosis factor α (*Tnf*) (n = 6–10/group, n = 3 Balb/c and NOD Chow (not detected in other mice for these strains)). (**I**) Plasma C-reactive protein (CRP) (n = 8–10/group), with mouse strains in all graphs sorted by increasing prevalence of NASH and liver fibrosis (as assessed by histopathological grading). Data are means ± SEM, *p < 0.05 vs. respective controls, as assessed by two-way unpaired t-test.

Consistent with the histopathology scoring, gene expression of α-1 type I collagen (*Col1a1*), the major component of fibrillar collagen in the liver, the profibrogenic cytokine transforming-growth factor-1 (*Tgfb1*) and tissue inhibitor of metalloproteinases 1 (*Timp1*), which inhibits matrix degradation, was increased in the four NASH susceptible strains (A/J, BL6, CBA and FVB/N–*Timp1* in FVB p = 0.065) but not in the NASH resistant mouse strains, except for a significant increase in *Timp1* gene expression in C3H mice with NASH (Fig. 2A–C). In contrast, expression of α-2 actin (*Acta2*), a marker of hepatic stellate cell (HSC) activation, was increased in C3H mice but reduced in A/J mice, while connective tissue growth factor (*Ccn2/Ctgf*) expression was unaffected by the NASH diet in all strains (Fig. 2D,E).

We next assessed gene expression of adhesion G protein-coupled receptor E1 (*Adgre1 / F4/80*), a widely used monocyte-macrophage marker, and found *F4/80* expression to be increased in NOD, A/J, BL6 and CBA mice (Fig. 2F). Gene expression of chemokine (C–C motif) ligand 2 (*Ccl2 / Mcp1*) and tumour necrosis factor-α (*Tnf*) were measured as readouts of hepatic inflammation. *Mcp1* expression was increased in the four NASH susceptible mouse strains (A/J, BL6, CBA and FVB/N) as well as in C3H mice (Fig. 2G), while *Tnfa* expression was increased in NOD, A/J, BL6 and CBA mice (Fig. 2H). We also assessed plasma C-reactive protein (CRP), which is used clinically as a biomarker of systemic inflammation^19^. Plasma CRP was increased in the four NASH susceptible mouse strains (A/J p = 0.054, BL6, CBA and FVB/N) and one NASH resistant mouse strain (BALB/c) (Fig. 2I).

Taken together, both histopathological grading and gene expression analysis demonstrate that A/J, CBA, BL6 and FVB/N are susceptible to mild liver fibrosis and NASH, with significant presence of hepatic steatosis, inflammation and hepatocyte ballooning. In contrast, BALB/c, C3H, NOD and DBA only show mild (or no) hepatic steatosis, with no evidence of NASH or liver fibrosis.

### NASH-susceptible mouse strains show increased hepatic cholesterol and diacylglycerol synthesis rates in the liver

**Figure 3:**
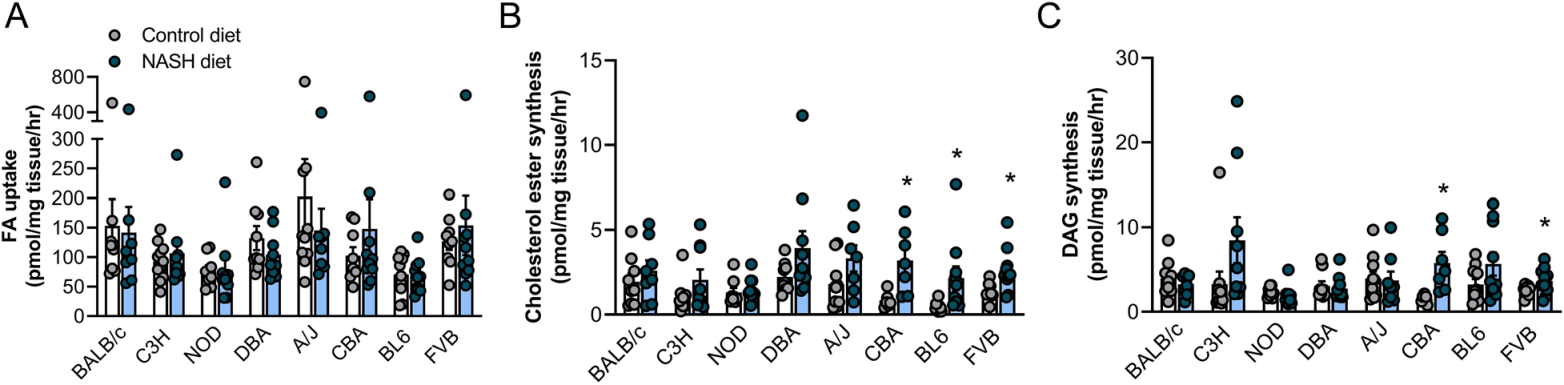
Assessment of hepatic lipid metabolism. Lipid metabolism was assessed in precision-cut liver slices using [^14^C]-radiolabelled fatty acids. (**A**) Fatty acid uptake (n = 8–11/group), (**B**) cholesterol ester synthesis (n = 6–11/group) and (**C**) diacylglycerol (DAG) synthesis (n = 6–10/group), with mouse strains in all graphs sorted by increasing prevalence of NASH and liver fibrosis (as assessed by histopathological grading). Data are means ± SEM, *p < 0.05 vs. respective controls, as assessed by two-way unpaired t-test.

NAFLD is associated with a multitude of defects in lipid metabolism that ultimately contribute to hepatic steatosis and NAFLD progression. These include, but are not limited to, increased fatty acid uptake and de novo lipogenesis, as well as a compensatory enhancement of fatty acid oxidation, which is insufficient to alleviate the lipid burden^20^. [^14^C]-tracer assays were used to assess hepatic lipid and glucose metabolism in precision-cut liver slices derived from mice. While fatty acid uptake was not impacted by the NASH diet in any mouse strain (Fig. 3A), cholesterol ester synthesis was significantly increased in the NASH susceptible CBA, BL6 and FVB/N strains (Fig. 3B), and diacylglycerol (DAG) synthesis was also increased in CBA and FVB/N mice (Fig. 3C). In contrast, the NASH diet did not affect fatty acid oxidation (Fig. S2A), triglyceride or ceramide synthesis, except for a significant 40% reduction in triglyceride synthesis in BALB/c mice and reduced ceramide synthesis in NOD and A/J mice (Fig. S2B,C). Phospholipid synthesis was only significantly increased in CBA mice with NASH (Fig. S2D), while neither glucose oxidation nor de novo lipogenesis were impacted by NASH in any mouse strain (Fig. S2E,F). Together, these data indicate modest changes in lipid metabolism in NASH susceptible mice, with increases in cholesterol ester synthesis being the dominant change.

### NASH-susceptible FVB/N mice show key features of the metabolic syndrome

**Figure 4:**
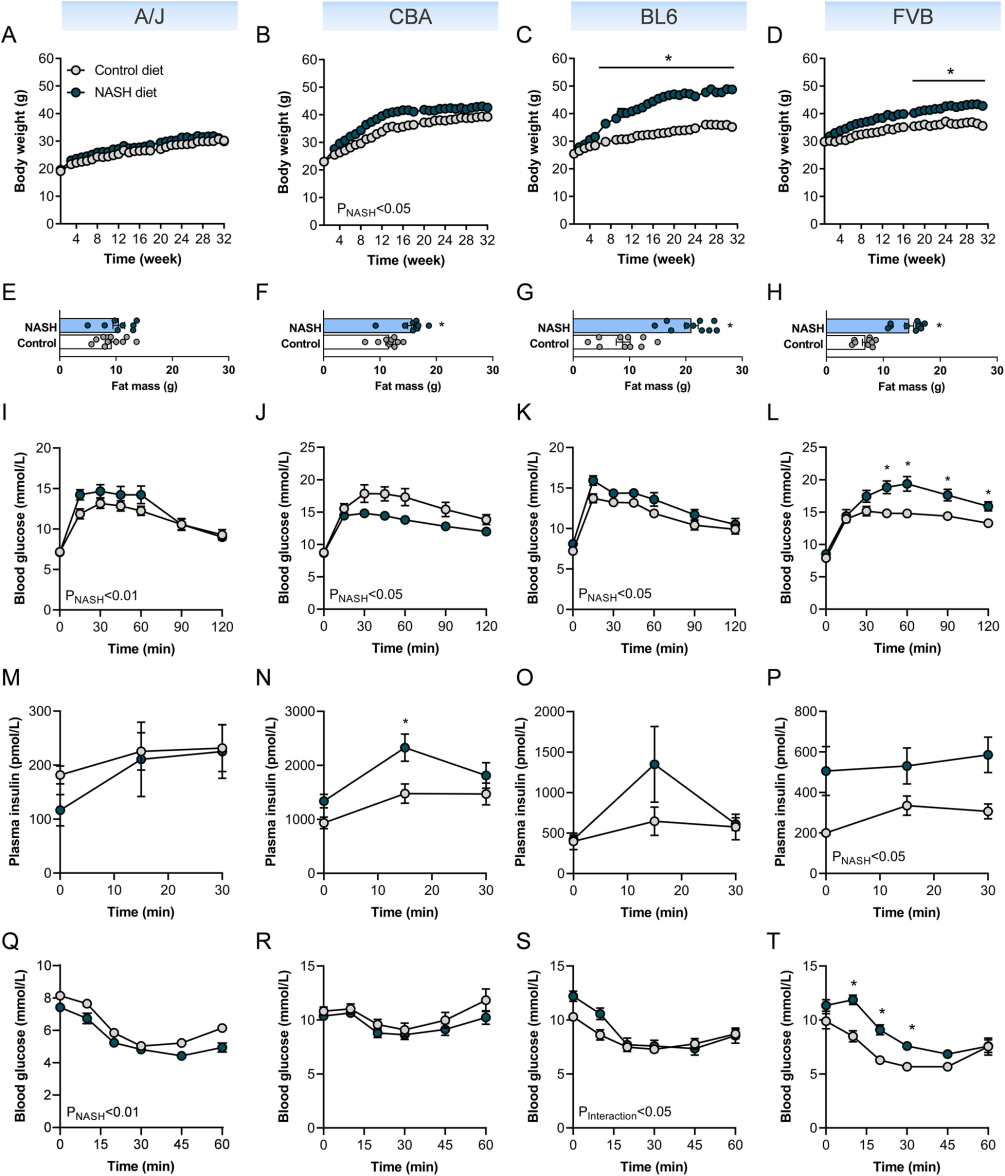
Metabolic phenotyping of NASH-susceptible mouse strains. Metabolic assessment is shown for the four NASH-susceptible mouse strains, including A/J, CBA/CaH, C57BL/6J and FVB/N, with mouse strains sorted by increasing prevalence of NASH and liver fibrosis (from left to right). (**A**–**D**) Weekly body weight (n = 8–10/group), (**E**–**H**) fat mass (n = 8–11/group), (**I**–**L**) glucose tolerance (n = 8–10/group), (**M**–**P**) plasma insulin assessed during the glucose tolerance test (n = 8–10/group), and (**Q**–**T**) insulin tolerance (n = 8–10/group). Data are means ± SEM, *p < 0.05 vs. respective controls, as assessed by two-way unpaired t-test (**E**–**H**), or two-way ANOVA and Bonferroni post-hoc analysis (**A**–**D**, **M**–**T**).

The histopathology analysis highlights that four of the eight mouse strains (A/J, CBA, BL6 and FVB/N) are susceptible to diet-induced NASH and moderate F1 liver fibrosis. We next assessed the presence of common NASH-associated metabolic comorbidities in mice. While metabolic assessments were conducted in all eight mouse strains, here we focus on the four NASH-susceptible strains. Data for the four NASH resistant strains are provided in Figs. S3 and S4. Of the NASH-susceptible mouse strains CBA, BL6 and FVB/N mice showed increased body weight and fat mass following the dietary intervention, while A/J mice were completely refractory to diet-induced obesity (Fig. 4A–H).

Further metabolic assessment showed that while A/J mice were resistant to diet-induced hyperglycaemia, hyperinsulinaemia (Table S4) and glucose-induced insulin secretion (Fig. 4M), they exhibited mild glucose intolerance (Fig. 4I), and even mild improvements in insulin sensitivity (Fig. 4Q, see Table S4 for glucose disappearance rate for ITT (KITT)). No differences were observed in the HOMA-IR used as insulin resistance index (Table S4). Thus, the A/J mouse strain does not develop metabolic co-morbidities associated with NASH.

Contrary to our expectations, CBA mice showed improvements in glucose tolerance when fed the NASH diet (Fig. 4J, see Table S4 for area-under-curve (AUC)), potentially related to substantial hyperinsulinaemia and in this respect increased HOMA-IR (Table S4), and increased glucose-induced insulin secretion (Fig. 4N), in the absence of fasting hyperglycaemia (Table S4) or insulin resistance (Fig. 4R, Table S4). While BL6 mice were resistant to fasting hyperglycaemia, hyperinsulinaemia (Table S4) and glucose-induced insulin secretion (Fig. 4O), with no change in the HOMA-IR (Table S4), they exhibited mild impairments in glucose tolerance (Fig. 4K, Table S4) and insulin sensitivity (Fig. 4S, Table S4). In contrast, while FVB mice did not develop hyperglycaemia (Table S4), they showed substantial glucose intolerance (Fig. 4L, Table S4), hyperinsulinaemia (Table S4) and increased glucose-induced insulin secretion (Fig. 4P). In addition, assessment of insulin tolerance showed that FVB mice were the only mouse strain that developed substantial insulin resistance (Fig. 4T, Table S4), with a significant increase in the HOMA-IR (Table S4). Taken together, these results indicate that from the four NASH susceptible mouse strains only FVB/N mice develop diet-induced obesity, glucose intolerance, hyperinsulinaemia and marked insulin resistance.

### All NASH-susceptible mouse strains exhibit hypercholesterinaemia

**Figure 5:**
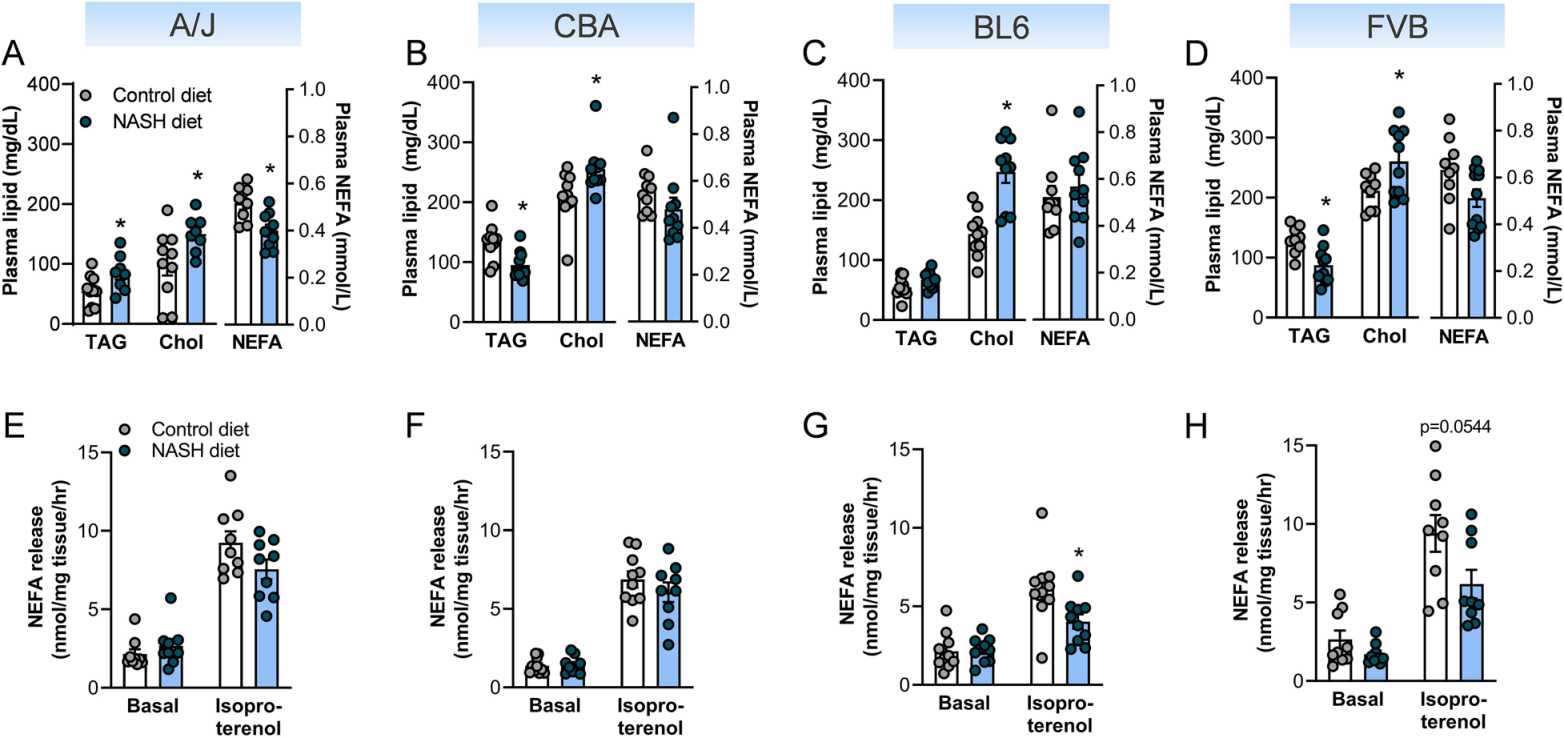
Assessment of plasma lipids and adipose tissue lipolysis in NASH-susceptible mouse strains. Metabolic assessment is shown for the four NASH-susceptible mouse strains, including A/J, C57BL/6J, CBA/CaH, and FVB/N, with mouse strains sorted by increasing prevalence of NASH and liver fibrosis (from left to right). (**A**–**D**) Plasma levels of triacylglycerol (TAG) (n = 6–10/group), total cholesterol (Chol) (n = 8–10/group) and non-esterified fatty acid (NEFA) (n = 8–10/group). (**E–H**) Assessment of NEFA release from epididymal adipose tissue explants as a readout of basal and isoproterenol-stimulated lipolysis (n = 8–10/group). Data are means ± SEM, *p < 0.05 vs. respective controls, as assessed by two-way unpaired t-test (**A**–**D**), or two-way ANOVA and Bonferroni post-hoc analysis (**E**–**H**).

In addition to impairments in glycaemic control and insulin action, NAFLD is associated with dyslipidaemia, including increased plasma triglyceride and low-density lipoprotein (LDL) cholesterol, and decreased high-density lipoprotein (HDL) cholesterol, with all being risk factors for cardiovascular disease^21^. Dietary cholesterol supplementation led to a significant increase in total plasma cholesterol in all four NASH-susceptible mouse strains. Plasma triglycerides were increased in A/J, were not different in BL6 mice and were surprisingly reduced in CBA and FVB/N mice (Fig. 5A–D). Plasma non-esterified fatty acids (NEFA) were reduced in A/J mice and were not different in the other NASH susceptible strains (Fig. 5A–D).

A major contributor to circulating NEFA, particularly in the fasted state, is the release of fatty acids from adipose tissue. Basal lipolysis is increased, and β-adrenergic stimulated lipolysis is decreased in obesity, which is linked to excessive ectopic lipid deposition and insulin resistance^22,23^. We therefore assessed lipolysis in epididymal adipose tissue explants in the basal state and following β-adrenergic stimulation (i.e., in the presence of isoproterenol). Basal lipolysis was unaffected by diet in any of the four NASH-susceptible mouse strains (Fig. 5E–H), while BL6 and FVB/N mice fed the NASH diet showed a significant reduction in β-adrenergic stimulated lipolysis (FVB p = 0.0544, Fig. 5E–H). Plasma lipids and lipolysis rates in the four NASH-resistant mouse strains (BALB/c, C3H, DBA and NOD) are shown in Fig. S4. Taken together, this analysis shows that FVB/N mice present with hypercholesterinaemia and mildly reduced catecholamine stimulation of lipolysis, in addition to obesity and impaired glycaemic control.

### NASH leads to inconsistent changes in kidney fibrosis

**Figure 6:**
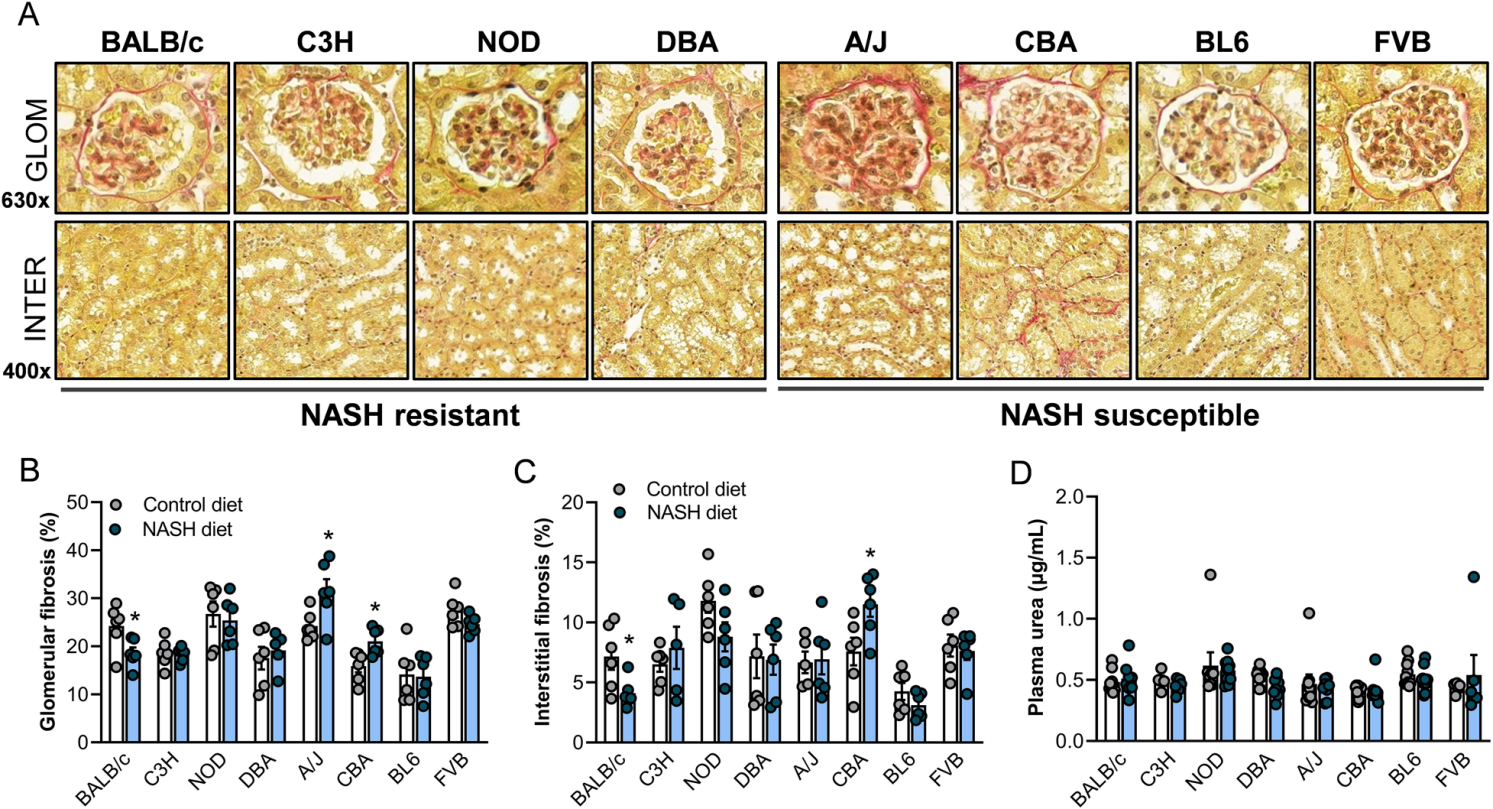
Assessment of renal fibrosis and plasma urea. (**A**) Representative renal glomerular and cortical interstitial histology (Picrosirius Red staining) in A/J, BALB/c, C3H/HeJ, C57BL/6J, CBA/CaH, DBA/2J, FVB/N and NOD/ShiLtJ mice fed a western-style diet, sorted by increasing prevalence of NASH and liver fibrosis. Percentage of glomerular (**B**) and interstitial (**C**) fibrosis (n = 5–6/group), and (**D**) plasma urea (n = 4–10/group) in all mouse strains. Data are means ± SEM, *p < 0.05 vs. respective controls, as assessed by two-way unpaired t-test.

An increasing body of evidence suggests that NAFLD and chronic kidney disease (CKD) share common pathogenetic mechanisms, and that NAFLD is related to the incidence and stage of CKD^24^. We therefore assessed changes in renal fibrosis, including glomerular and interstitial fibrosis, in all mouse strains using Picrosirius Red staining of coronal kidney histological sections (Fig. 6A, Fig. S5). While glomerular fibrosis was significantly increased in A/J and CBA mice (Fig. 6B) and CBA mice further showed increased interstitial fibrosis (Fig. 6C), BALB/c mice surprisingly exhibited reduced glomerular and interstitial fibrosis with the NASH diet (Fig. 6B,C). Furthermore, toxic metabolites that are usually eliminated by the kidneys, including urea, accumulate in the blood in CKD, and are useful markers of kidney dysfunction^25^. However, plasma urea levels were not impacted by the NASH diet in any of the mouse strains (Fig. 6D). Overall, these results highlight that while early-stage NASH is unlikely to be associated with severe impairments in renal function, CBA mice could be a useful model of western diet-induced renal fibrosis.

### NASH increases systemic fat oxidation

Obesity and metabolic disease are associated with changes in systemic energy metabolism and substrate utilization, including increased preference for fatty acid oxidation^26^. While ANCOVA analysis using (lean body mass + 0.2 × fat mass) as a covariate^27^ indicated that systemic energy expenditure was not impacted by NASH in any mouse strain (Fig. S6A), all eight mouse strains showed a significant reduction in the respiratory exchange ratio (Fig. S6B), demonstrating increased oxidation of fatty acids. Food intake was increased in C3H mice, but not impacted in any other strain (Fig. S6C), while locomotor activity was not impacted by the NASH diet in any mouse strain (Fig. S6D). Taken together, these data highlight that NASH induces a preferential oxidation of fatty acids in the absence of changes in systemic energy expenditure.

## Discussion

**Figure 7:**
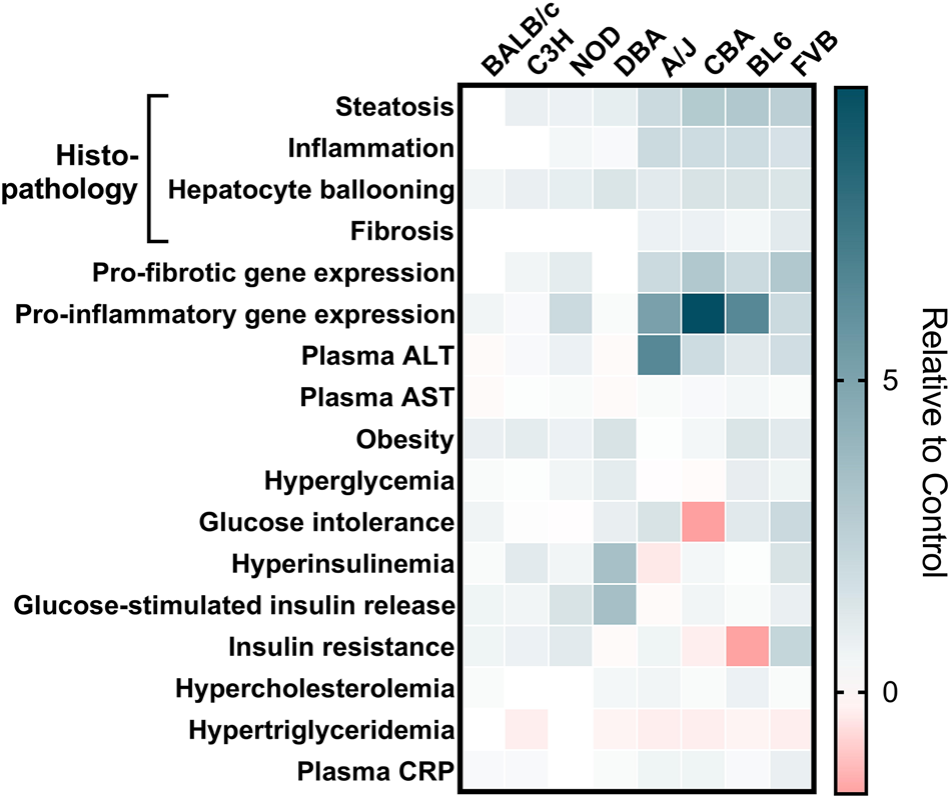
Summary heat map analysis highlighting the primary hepatic and metabolic changes observed in mice fed the NASH diet compared to their respective Controls, with all data points being relative and not representing actual values/data points. This heat map was considered for decision making of a ‘good’ NASH model. There was no formal weighting given to the criteria, with histological features of NASH as the primary consideration, then placing equal weighting on the metabolic comorbidities as a secondary consideration. For pro-fibrotic and pro-inflammatory gene expression, all variables measured through gene expression received equal weighting. Glucose intolerance is shown as total AUC, while insulin resistance is shown as glucose disappearance rate for the ITT (KITT).

Despite liver fibrosis being a strong contributor to liver-related and overall mortality^5^, there are currently no approved pharmacotherapies for inhibiting or reversing progression of NASH and liver fibrosis. A major obstacle for successful translation of findings from animal models to humans is the lack of adequate mouse models that recapitulate human NASH pathology and the accompanying metabolic comorbidities. Through deep phenotypic assessment, we show that FVB/N mice fed a western diet recapitulate the key features of human NASH (Fig. 7) and advocate their use as a suitable pre-clinical model to assess therapeutic strategies for NASH with mild liver fibrosis. With efforts in the pharmaceutical industry now focussed on developing multi-faceted therapies; that is, therapies that improve NASH and/or liver fibrosis, and concomitantly treat other metabolic comorbidities, this mouse model is well suited for such pre-clinical use.

Numerous mouse models of NASH have been developed, including various dietary approaches, as well as toxins (e.g., carbon tetrachloride, streptozotocin) or genetic manipulation in the absence/presence of dietary interventions. The majority of such studies utilize the C57BL/6 (BL6) mouse strain, due to their availability and low cost, detailed phenotypic characterization, and overall favoured standing as the background strain for most genetically engineered models^12^. In these BL6-centered studies, mouse models of NASH can be broadly subdivided into (i) severe NASH and liver fibrosis in the absence of features of the metabolic syndrome (e.g., methionine choline deficient (MCD) diet, SREBP1c TG or PPARα^-/-^), or (ii) hepatic steatosis with presence of metabolic dysfunction but in the absence of NASH and / or liver fibrosis (e.g., high-fat diet (HFD), *ob/ob*)^13^. A relatively new model is the MUP-uPA transgenic mouse line, which expresses transiently high amounts of urokinase plasminogen activator in hepatocytes, and develops both NASH pathology and extensive metabolic dysfunction when fed a HFD. However, the majority of these mice spontaneously progress to developing hepatocellular carcinoma, thereby precluding longer-term examination of NASH / fibrosis^13^. Here, we show that while BL6 mice fed a western diet enriched in fat, sucrose, fructose and cholesterol develop NASH with mild liver fibrosis, they exhibit very mild glucose intolerance and insulin resistance, with no changes in fasting blood glucose or plasma insulin, or glucose-stimulated insulin secretion. Typically, metabolic impairments in mice are assessed with limited temporal resolution, most commonly following a 8–12 week dietary intervention, pointing to severe hyperinsulinemia and impairments in glycaemic control in the BL6 mouse strain^28^, as also frequently reported by our group^26,29^. However, recent studies show that while prolonged exposure to high-fat high-sugar diets leads to morbid obesity and NAFLD, changes in glucose homeostasis can be highly dynamic, with glucose intolerance and insulin sensitivity initially deteriorating but being indistinguishable to that of chow mice following > 24 weeks of high-fat feeding^30,31^, as is the case in our study. In previous studies, this time-dependent improvement in glucose homeostasis coincided with adaptive β-cell hyperplasia^30^ or a preservation of peripheral glucose disposal^31^. While hyperinsulinemia was not affected in BL6 mice fed a western diet, we did not have the capacity to assess the contribution of peripheral tissues to the blood glucose response.

In-depth assessment of NASH histopathology and extensive metabolic phenotyping identified the FVB/N mouse strain as the optimal diet-induced mouse model for NASH, mild F1 liver fibrosis and a broad spectrum of metabolic comorbidities, most prominently presence of obesity, glucose intolerance, hyperinsulinemia, insulin resistance, hypercholesterinemia, systemic inflammation, and a mild impairment in catecholamine regulation of adipose tissue lipolysis. Our histopathological and metabolic assessment of FVB/N mice agrees with previous reports indicating that FVB/N mice are highly susceptible to HFD-induced obesity, glucose intolerance, insulin resistance and hepatic steatosis^26,33^. Similarly, *ob/ob* mice bred on an FVB/N genetic background show more severe hyperglycaemia and hepatic insulin resistance when compared to *ob/ob* on a C57BL/6 J background^34^. FVB/N mice show high incidence of spontaneous multifocal hepatocellular necrosis^35^, and exhibit the greatest increase in liver weight, prevalence of periductal fibrosis, highest rates of plasma ALT and substantial hepatocyte ballooning compared to four other mouse strains, when administered an agent that induces Mallory-Denk bodies (i.e., hepatocyte inclusions found in several liver diseases that correlate with hepatocyte ballooning)^36^, overall indicating that FVB/N mice have a high predisposition to advanced liver disease. Given that ~ 85% of NASH patients are overweight or obese, ~ 50% have impaired glucose tolerance and/or hyperinsulinemia, 98% have insulin resistance and 72% have dyslipidaemia^6,37^, the metabolic defects in FVB/N mice are representative of the most common metabolic defects observed in human NASH. While FVB/N mice only develop mild F1 fibrosis, their liver phenotype is similar to the vast majority of human NAFLD patients, where on average 12% and 5% of obese patients have NASH and fibrosis, respectively, and importantly, of those with fibrosis, 83% have F1 fibrosis^38^. Therefore, this mouse model is of relevance to testing therapeutics that would be useful for early prevention and would benefit the majority of NASH patients. However, it should be noted that while ~ 80% of NASH patients also present with hypertriglyceridemia^6,37^, plasma triglyceride levels were reduced in FVB/N mice fed the NASH diet, potentially related to reduced VLDL secretion, as dysfunctional VLDL secretion has previously been described as a feature of NASH^39^.

CBA/CaH mice also developed NASH and hepatic F1 fibrosis, as well as renal fibrosis, however these mice did not develop insulin resistance, and exhibited improvements in glucose tolerance, which was likely related to their considerable hyperinsulinemia. These observations contrast previous studies suggesting that 10–20% of CBA/Ca mice are prone to ‘spontaneous maturity onset diabetes obesity syndrome’, which includes presence of obesity, hyperglycaemia, impaired glucose tolerance and hyperinsulinaemia^40,41^. In addition, our macroscopic evaluation of CBA livers suggested that 30% of mice developed hepatocellular carcinoma, which is in accordance with previous studies reporting high tumour incidence in CBA mice^42^. Despite their susceptibility to liver cancer and metabolic disease in a subset of mice, little is known about spontaneous and diet-induced predisposition to NAFLD in this mouse strain. The present results showing the absence of hyperglycaemia, glucose intolerance and insulin resistance, indicate that this strain is limited as a pre-clinical model for metabolic (dysfunction) associated fatty liver disease (MAFLD).

While BL6 and FVB/N mice developed many metabolic defects with western diet feeding, the A/J mouse strain, which also developed NASH and F1 fibrosis, was completely refractory to diet-induced obesity and various metabolic defects, including hyperglycaemia and hyperinsulinemia, and exhibited only very mild glucose intolerance and even mild improvements in insulin sensitivity. While the A/J strain is known to be resistant to diet-induced obesity^43,44^, the presence of NASH and fibrosis observed in our study was surprising given that previous studies suggested that A/J mice are resistant to NAFLD in the context of diet-induced obesity^12^. However, it should be noted that hepatic triglyceride levels in A/J NASH mice were ~ threefold lower than in western diet-fed BL6, CBA and FVB/N mice, and were overall more similar to the four NASH-resistant mouse strains, highlighting their relative resistance to hepatic lipid accumulation. Previous studies in A/J mice employed high-fat and high-sucrose dietary interventions, whereas the western diet used in the present study was further enriched in cholesterol and fructose, a carbohydrate known to drive hepatic lipogenesis^29^.

While this study focussed primarily on the identification of NASH susceptible mice with metabolic comorbidities, we further identified the BALB/c mouse strain as completely refractory to hepatic steatosis, NASH and liver fibrosis, in fact, these mice even showed reduced renal fibrosis despite the presence of obesity, hyperinsulinemia and hypercholesterinaemia. We have previously shown that BALB/c mice are resistant to both lipid- and fructose-induced hepatic steatosis^26,29^, and are refractory to fructose-induced activation of lipogenic pathways in the liver^29^, which is likely to contribute to their resistance to the development of metabolic dysfunction when fed a western diet. The complete lack of diet-induced hepatic deterioration is intriguing, and understanding the molecular pathways underlying such resistance to NAFLD may lead to the identification of novel therapeutic strategies for NASH and liver fibrosis.

In addition to detailed hepatic histopathological analysis, we provide in-depth assessment of diet-induced changes in hepatic lipid and glucose metabolism using carbon-14 tracer studies in precision-cut liver slices. We observed increased rates of DAG and cholesterol ester synthesis in the NASH sensitive CBA and FVB/N mice, as well as increased cholesterol ester synthesis in BL6 mice. Disturbances in cholesterol metabolism, particularly an increase in cholesterol synthesis and accumulation of free cholesterol, contribute to the pathophysiology of NASH by driving hepatic inflammation and fibrosis^45,46^, and are likely to contribute to the susceptibility of BL6, CBA and FVB/N mouse strains to NASH. In addition, the increase in DAG synthesis is likely to further contribute to disease progression in CBA and FVB/N mice being an intermediate in TAG synthesis and a direct inhibitor of hepatic insulin action through activation of protein kinase c signalling^47^. In contrast, fatty acid uptake, fatty acid and glucose oxidation, and de novo lipogenesis were not impacted by NASH in these strains. This was surprising given that NAFLD/NASH is commonly associated with increased lipogenesis and fatty acid uptake, as well as reduced β-oxidation capacity and mitochondrial dysfunction^48,49^, particularly with dietary fructose driving lipogenesis and suppressing fat oxidation^50^. Interestingly, BALB/c mice showed a significant reduction in triglyceride synthesis when fed the NASH diet, which may contribute to their resistance to hepatic steatosis and NASH. While BALB/c mice are known to be completely refractory to fructose-induced activation of lipogenesis^29^, the overall discrepancies are potentially related to the lack of hormonal regulation in the ex vivo liver slice experimental system, and the lack of an integrated in vivo response, including absence of hyperglycaemia and hyperinsulinemia, with glucose and insulin well known to contribute to excessive lipid accumulation^51^.

In conclusion, we provide a comprehensive assessment of liver pathology and the metabolic phenotype of eight commonly used inbred mouse strains, and show that FVB/N is the mouse strain that most faithfully recapitulates human NASH pathophysiology, including the most predominant NASH-associated metabolic comorbidities. The mild induction of F1 fibrosis is representative of the vast majority of human NASH patients, therefore providing a useful model for assessment of novel therapies for ‘borderline’ NASH and prevention of disease progression. A longer feeding regime and/or modulation of diet composition might provide a better model for advanced NASH, more severe liver fibrosis and potentially hepatocellular carcinoma, while a more in-depth metabolic analysis on these mice, including measures of adipose tissue inflammation and heart function, would likely provide further insights into the suitability of the FVB/N strain as a useful model for MAFLD. Finally, future studies in female mice are warranted given the equally high incidence of NASH in males and females.

## Methods and materials

### Animal studies

Eight common in-bred mouse strains, including A/J, BALB/c, C3H/HeJ, C57BL/6J, CBA/CaH, DBA/2J, FVB/N and NOD/ShiLtJ, were sourced from the Animal Resources Centre (Canning Vale, Australia), and will be referred to as A/J, BALB/c, C3H, BL6, CBA, DBA, FVB and NOD throughout the manuscript. Male mice were housed at 22 °C on a 12:12-h light–dark cycle and fed either a rodent chow diet or a western-style diet enriched in fat (39.8% energy from fat; of this 18% trans-fat), carbohydrates (40% energy from carbohydrate; of this 30% fructose), protein (20% energy from protein) and 2% cholesterol (20 g/kg) (SF16-033, Specialty Feeds, Australia), from now on referred to as NASH diet. The chow diet is a cereal grain-base pellet diet provided in the form of 12 mm pellets, containing 5% energy from fat, while the NASH diet is a semi-pure high fat diet formulation based on Research Diets D09100301. The NASH diet contains a fixed formula ration using the following ingredients: wheat, barley, lupins, soya meal, fish meal, mixed vegetable oils, canola oil, salt, calcium carbonate, dicalcium phosphate, magnesium oxide, and a vitamin and trace mineral premix (see Table S1 for full diet composition). Mice were fed the NASH diet from 8–9 weeks of age for a total of 30–32 weeks. Prior to commencing the experimental diet, mice were matched by body weight within their strain and grouped into NASH (n = 8–10) and control groups (n = 8–10), and body weight was recorded weekly during the feeding regime. Experimental procedures were approved by the University of Melbourne Anatomy & Neuroscience, Pathology, Pharmacology, and Physiology Animal Ethics Committee (ethics application No. 2015115) and conformed to the National Health and Medical Research Council of Australia guidelines regarding the care and use of experimental animals, and followed the recommendations in the ARRIVE guidelines^52^.

### Body composition and energy expenditure

Fat and lean mass were measured using the Bruker LF50H Minispec Body Composition Analyser (Coherent Scientific Pty Ltd, Thebarton, Australia), in accordance with the manufacturer’s instructions. Whole-body energy expenditure, the respiratory exchange ratio, food intake and physical activity were assessed using a 16-chamber Promethion Metabolic System (Sable, Nevada, USA). Studies were commenced 24 h after acclimatisation and the above parameters were monitored thereafter for a further 24 h in 30 min intervals.

### Glucose and insulin tolerance

Mice were fasted for 4 h starting at 7:00, gavaged with glucose (2 g/kg body weight) or injected i.p. with insulin (0.75 U/kg body weight, Actrapid), and blood glucose levels were assessed (Accu-Chek II glucometer; Roche Diagnostics, Castle Hill, Australia) before and at selected time points after glucose/insulin administration. During the glucose tolerance test, additional blood was collected for assessment of plasma insulin by ELISA (Ultra-Sensitive Mouse Insulin ELISA; Crystal Chem, Elk Grove Village, IL, USA).

### Liver histopathology

A piece of liver consistently obtained from the same left lobe was fixed in 10% formalin, embedded in paraffin, and 5 μm sections were cut and stained with Masson Trichrome (MT), hematoxylin–eosin (H&E) or Picrosirius red (PR) by Phenomics Australia Histopathology and Slide Scanning Service (University of Melbourne, Australia). Histopathological analysis was conducted and scored by a single pathologist blinded to mouse strain and dietary intervention, in accordance with the Clinical Research Network (CRN) NAFLD activity score (NAS)^53^ and Kleiner classification of liver fibrosis^54^. Samples were stratified according to hepatic scores for steatosis (grade 1, 5–33% of parenchyma; grade 2, > 33–66% of parenchyma; grade 3, > 66% of parenchyma), inflammation (grade 1, < 2 inflammatory foci per × 200 field; grade 2, 2–4 foci; grade 3, > 4 foci), hepatocyte ballooning (few or many ballooning cells are present per high-power field for grade 1 or 2, respectively) and fibrosis (grading according to Kleiner^54^). Presence of NASH was determined as joint presence of steatosis, hepatocyte ballooning and lobular inflammation (NAS ≥ 3), as defined by the Clinical Practice Guidelines of European Association for the Study of the Liver (EASL), the European Association for the Study of Diabetes (EASD) and European Association for the Study of Obesity (EASO)^55^. Percentage fibrosis area was calculated using Orbit Image Analysis^56^ and five representative PR images across each liver section at 100 × magnification.

### Kidney histopathology

The left kidney from each mouse was fixed in 10% formalin, embedded in paraffin, 5 μm coronal sections were cut and stained with Picrosirius red (PR) by Phenomics Australia Histopathology and Slide Scanning Service (University of Melbourne, Australia). Percentage fibrosis area was calculated using Orbit Image Analysis^56^, using either four representative glomeruli from each section at 630 × magnification, or two representative PR images of the cortical interstitium at 400 × magnification.

### Hepatic triglyceride content

Total liver triglyceride content was determined by mass spectrometry. Briefly, liver (~ 6 mg) was homogenized in 100 μl 1:1 butanol-methanol (v/v), containing 5 μL of SPLASH® II LIPIDOMIX® Mass Spec Standard (330709W, Avanti Polar Lipids Inc.) using a Precellys Evolution Tissue Homogenizer (Bertin Instruments, USA). Samples were mixed thoroughly for 1 h at room temperature, centrifuged (14,000*g*, 10 min, 20 °C) and transferred into sample vials with glass inserts for analysis by ultrahigh performance liquid chromatography (UHPLC) coupled to tandem mass spectrometry (MS/MS) employing a Vanquish UHPLC linked to an Orbitrap Fusion Lumos mass spectrometer (Thermo Fisher Scientific).

### Assessment of hepatic lipid and glucose metabolism

Lipid metabolism was assessed using freshly prepared 300 μm precision-cut liver slices. Briefly, a piece of liver obtained from the same lobe of the liver for each mouse was embedded in 3% agarose (SeaPlaque™ Agarose, Lonza Bioscience) and 300 µm thick liver slices were generated using a Krumdieck Tissue Slicer (Alabama Research and Development). Liver slices were settled in oxygenated M199 media (Thermo Fisher Scientific, Scoresby, Australia) for 60 min before metabolic assessment. For assessment of fatty acid metabolism, liver slices were then incubated in 1 mL of low glucose DMEM (Thermo Fisher Scientific, Scoresby, Australia) containing 2% BSA (w/v), 500 μmol/L oleate and 1 μCi/mL [1-^14^C] oleate for 2 h. At the completion of the experiment, the culture medium was acidified in 1 mL 1 mol/L perchloric acid to liberate ^14^CO_2_ derived from complete fatty acid oxidation, which was collected in 300 μL 1 mol/L NaOH and counted on a Tri Carb 2810TR liquid scintillation analyser (Perkin Elmer, Massachusetts, USA). The liver slices were washed in PBS and homogenized in 1 mL 2:1 chloroform:methanol (v:v) to extract lipids. The homogenate was centrifuged at 2000*g* for 10 min to achieve phase separation, the upper aqueous phase containing acid-soluble β-oxidation intermediates (ASM) was counted using the liquid scintillation analyser, the lower organic phase was transferred to a fresh tube, dried under N_2_ at 40 °C, and reconstituted in 2:1 chloroform : methanol containing the following lipid standards: cholesterol linoleate (C0289, Sigma-Aldrich, St. Louis, MO, USA), glyceryl tripalmitate (T5888, Sigma-Aldrich), oleate (O1008, Sigma-Aldrich), dipalmitin (D2636, Sigma-Aldrich), C24:0 ceramide (43799, Sigma-Aldrich) and l-α-phosphatidylcholine (P2772, Sigma-Aldrich). The lipid mixtures were spotted onto glass-backed Silica Gel 60 plates and the lipids were resolved in a 65:25:4 chloroform:methanol:water (v/v) solution, followed by two runs in 75:35:1 hexane:diethyl ether: acetic acid (v/v). The plates were air-dried, sprayed with dichlorofluorescein (0.02% w/v in ethanol) dye and the lipid bands were visualized under UV light. The lipid bands were scraped, and radioactivity measured on the liquid scintillation counter. Total fatty acid oxidation was calculated as the sum of radioactivity detected in the CO_2_ and ASM fractions, while fatty acid uptake was calculated as the sum of total fatty acid oxidation, and radioactivity detected in all lipid fractions. All values were normalized to liver slice weight.

Glucose oxidation and lipogenesis were measured for 2 h in low glucose DMEM (Thermo Fisher Scientific, Scoresby, Australia) containing 2% BSA and 1 μCi/mL U-^14^C-glucose (Perkin Elmer). Similar to above, the media was acidified to liberate and capture ^14^CO_2_ as a readout of glucose oxidation, while liver slices were washed, lipids extracted and radioactivity within the total lipid extract counted for assessment of lipogenesis.

### Adipose tissue lipolysis

Lipolysis was assessed as the release of non-esterified fatty acids (NEFA) from epididymal adipose tissue explants in the basal state and following β-adrenergic stimulation. Briefly, adipose tissue was incubated in phenol red-free low glucose DMEM (Thermo Fisher Scientific, Scoresby, Australia) containing 2% BSA, in the absence or presence of isoproterenol (1 µM) for 2 h. NEFA release into the media was assessed by colorimetric assay (Wako HR series NEFA-HR(2), Osaka, Japan).

### Plasma analysis

Plasma was collected following a 4 h fast and used for assessment of triglycerides (Triglycerides GPOPAP; Roche Diagnostics, Indianapolis, IN), NEFA (Wako Pure Chemical Industries, Osaka, Japan), cholesterol (Abcam, ab65390), C-reactive protein (Mouse C-Reactive Protein (CRP) ELISA Kit; Crystal Chem, Elk Grove Village, IL, USA), urea (Urea Assay Kit, Cell Biolabs Inc, BioAssay Systems), as well as alanine aminotransferase (ALT) and aspartate aminotransferase (AST) activity, as described previously^57,58^.

### Gene expression analysis

Total RNA was extracted from liver using TRI reagent (Sigma Aldrich, Castle Hill, Australia), treated with DNase (Ambion DNA free kit, Thermo Fisher, VIC, Australia) and reverse transcribed using iScript Reverse Transcriptase (Invitrogen, USA). Real-time PCR was performed using SYBR Green PCR Master Mix (Quantinova® SYBR Green PCR kit, QIAGEN; Germany) on a CFX Connect™ Real-Time PCR Detection System (Bio-Rad Laboratories, Gladesville, Australia). Samples were normalized to *Hprt* as a housekeeping gene. Primer sequences are provided in Table S2.

### Statistical analysis

Data are presented as mean ± standard error of the mean (SEM), with statistical significance set at the 5% level of significance (P < 0.05). Data were assessed for normal distribution using the D’Agostino and Pearson test with parametric tests used in cases of normal distribution, and non-parametric tests used in cases of non-normal distribution. Parametric tests included, where appropriate, a two-tailed unpaired Students t-test, one or two-way analysis of variance (ANOVA) followed by Bonferroni multiple comparisons tests. Non-parametric testing included the Wilcoxon matched pairs signed rank test. The energy expenditure ANCOVA analysis done for this work was provided by the NIDDK Mouse Metabolic Phenotyping Centers (MMPC, www.mmpc.org) using their Energy Expenditure Analysis page (http://www.mmpc.org/shared/regression.aspx) and supported by grants DK076169 and DK115255.

## Supplementary Information

Supplementary Information.

## Data Availability

All data analysed during this study are included in the published article (and its online supplementary files). No applicable resources were generated or analysed during the current study.
