# Supplementary material for: Mouse strain-dependent variation in metabolic associated fatty liver disease (MAFLD): a comprehensive resource tool for pre-clinical studies
Source: Sci Rep. 2023 Mar 22;13:4711. doi: 10.1038/s41598-023-32037-1 (PMC10033881; doi:10.1038/s41598-023-32037-1)
Supplement: Supplementary file 1 — Supplementary Information. [file 41598_2023_32037_MOESM1_ESM.pdf]

Mouse strain-dependent variation in metabolic associated fatty liver disease (MAFLD) – A comprehensive resource tool for pre-clinical studies

Authors: Hamzeh Karimkhanloo[1, 2], Stacey N. Keenan[1], Jacqueline Bayliss[1], William De Nardo[1], Paula M. Miotto[1], Camille Devereux[1], Shuai Nie[3], Nicholas A. Williamson[3], Andrew Ryan[4], Matthew J. Watt[1*], Magdalene K. Montgomery[1*]

Affiliations:

[1] Department of Anatomy and Physiology, University of Melbourne, Melbourne, VIC 3010, Australia

[2] Metabolism, Diabetes and Obesity Program, Monash Biomedicine Discovery Institute, and Department of Physiology, Monash University, Clayton, Victoria, Australia

[3] Melbourne Mass Spectrometry and Proteomics Facility, Bio21 Molecular Science & Biotechnology Institute, The University of Melbourne, Melbourne, Victoria, Australia.

[4] TissuPath, Mount Waverley, VIC 3149, Australia.

Figure S1

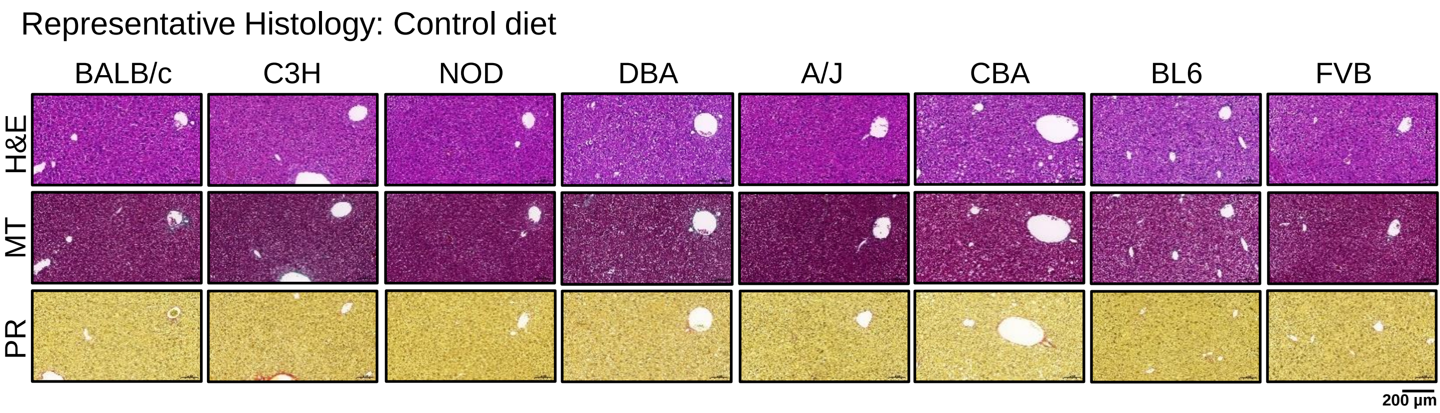

Figure S1. Liver histopathology staining in all mouse strains fed a Control diet. Representative liver histology (H&E, Masson's Trichrome and Picrosirius Red staining) in A/J, BALB/c, C3H/HeJ, C57BL/6J, CBA/CaH, DBA/2J, FVB/N and NOD/ShiLtJ mice fed a Chow diet, sorted by increasing prevalence of NASH and liver fibrosis (in the respective western diet-fed mice).

Figure S2

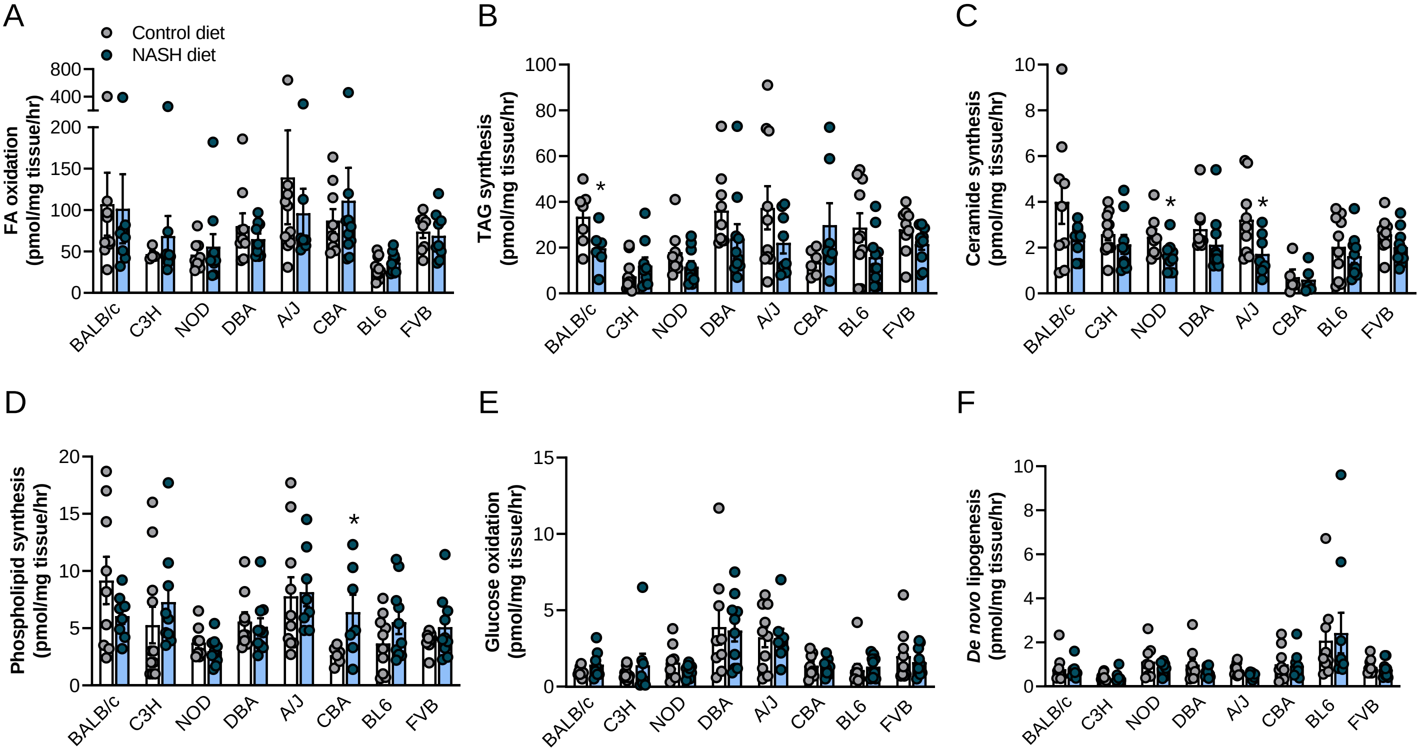

Figure S2. Assessment of hepatic lipid and glucose metabolism. Lipid and glucose metabolism was assessed in precision-cut liver slices using [^{14}C]-radiolabelled fatty acids or glucose, respectively. **(A)** Fatty acid oxidation (n=8-10/group, n=4 C3H Chow), **(B)** triglyceride synthesis (n=6-11/group), **(C)** ceramide synthesis (n=5-11/group), **(D)** phospholipid synthesis (n=6-11/group), **(E)** glucose oxidation (n=8-11/group), and **(F)** *de novo* lipogenesis (n=8-11/group). Data are means ± SEM, * p<0.05 vs. respective controls, as assessed by two-way unpaired t-test.

Figure S3

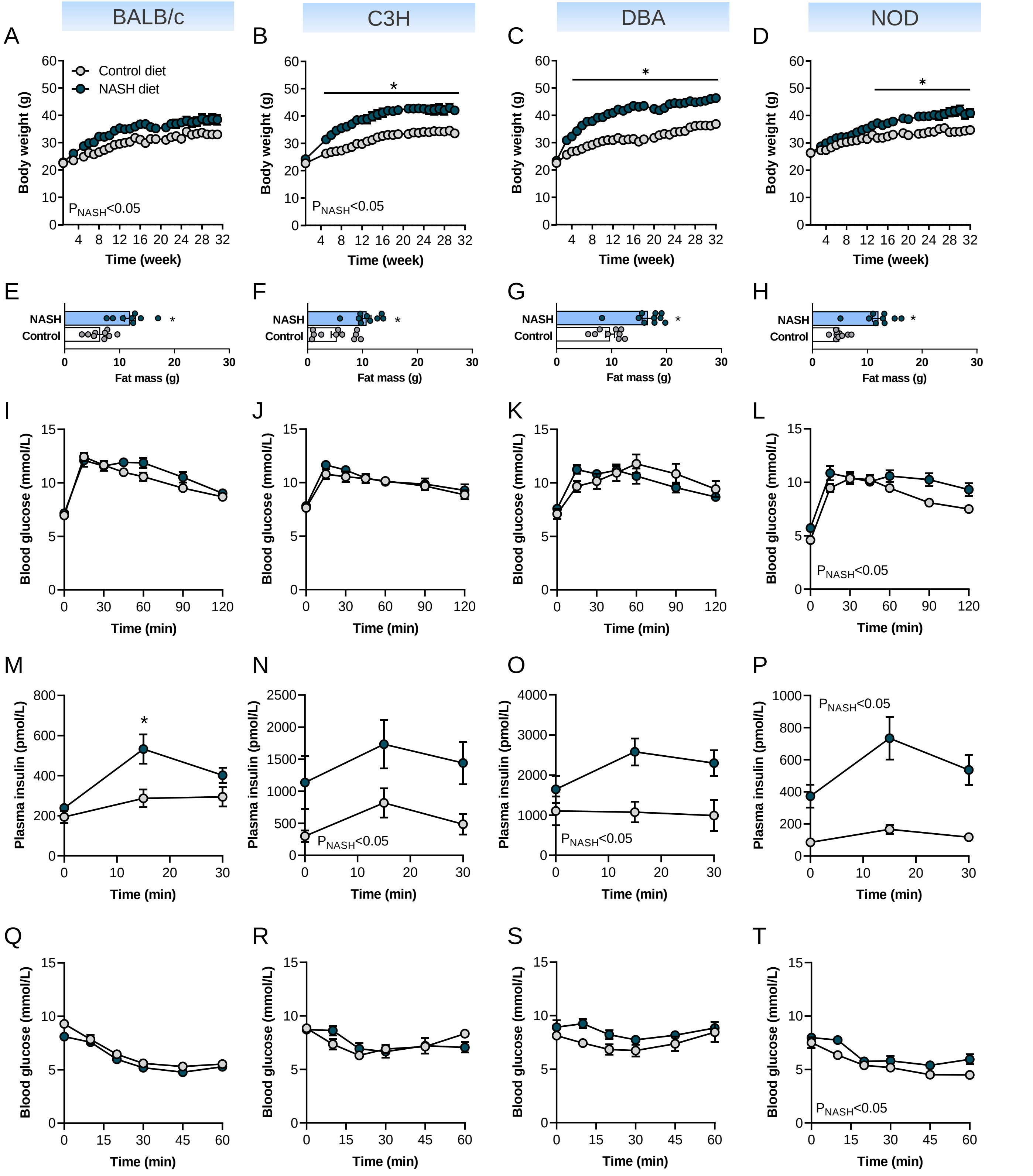

Figure S3. Metabolic phenotyping of NASH-resistant mouse strains. Metabolic assessment is shown for the four NASH-resistant mouse strains, including BALB/c, C3H/HeJ, DBA/2J and NOD/ShiLtJ, with mouse strains sorted by increasing prevalence of NAFLD (from left to right). **(A-D)** Weekly body weight (n=8-11/group), **(E-H)** fat mass (n=8-11/group), **(I-L)** glucose tolerance (n=8-10/group), **(M-P)** plasma insulin assessed during the glucose tolerance test (n=8-10/group), and **(Q-T)** insulin tolerance (n=8-10/group). Data are means ± SEM, * $p<0.05$ vs. respective controls, as assessed by two-way unpaired t-test (E-H), or two-way ANOVA and Bonferroni post-hoc analysis (A-D, M-T).

Figure S4

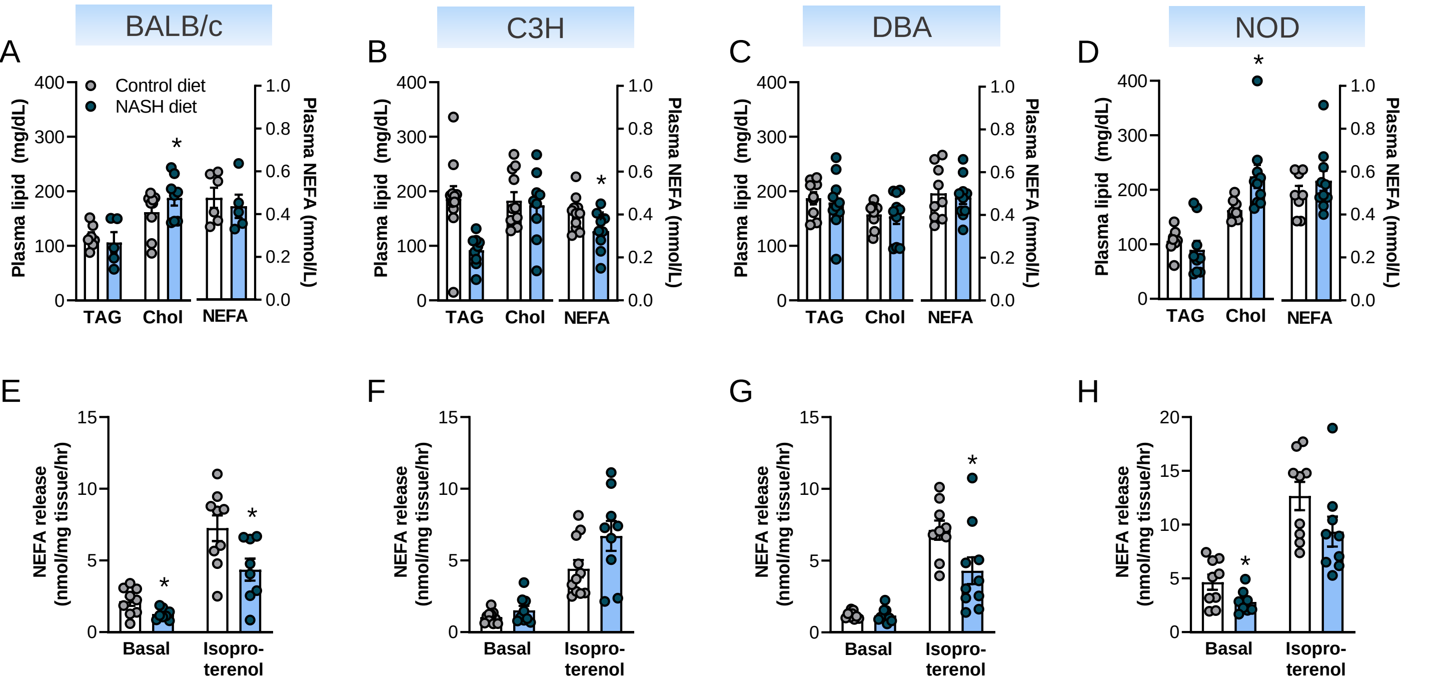

Figure S4. Assessment of plasma lipids and adipose tissue lipolysis in NASH-resistant mouse strains. Metabolic assessment is shown for the four NASH-resistant mouse strains, including BALB/c, C3H/HeJ, DBA/2J and NOD/ShiLtJ, with mouse strains sorted by increasing prevalence of NAFLD (from left to right). **(A-D)** Plasma levels of triacylglycerol (TAG) (n=6-10/group, n=4 C3H Chow), total cholesterol (Chol) (n=8-11/group) and non-esterified fatty acid (NEFA) (n=5-10/group). **(E-H)** Assessment of NEFA release from epididymal adipose tissue explants as a readout of basal and isoproterenol-stimulated lipolysis (n=8-10/group). Data are means ± SEM, * $p<0.05$ vs. respective controls, as assessed by two-way unpaired t-test (A-D), or two-way ANOVA and Bonferroni post-hoc analysis (E-H).

Figure S5

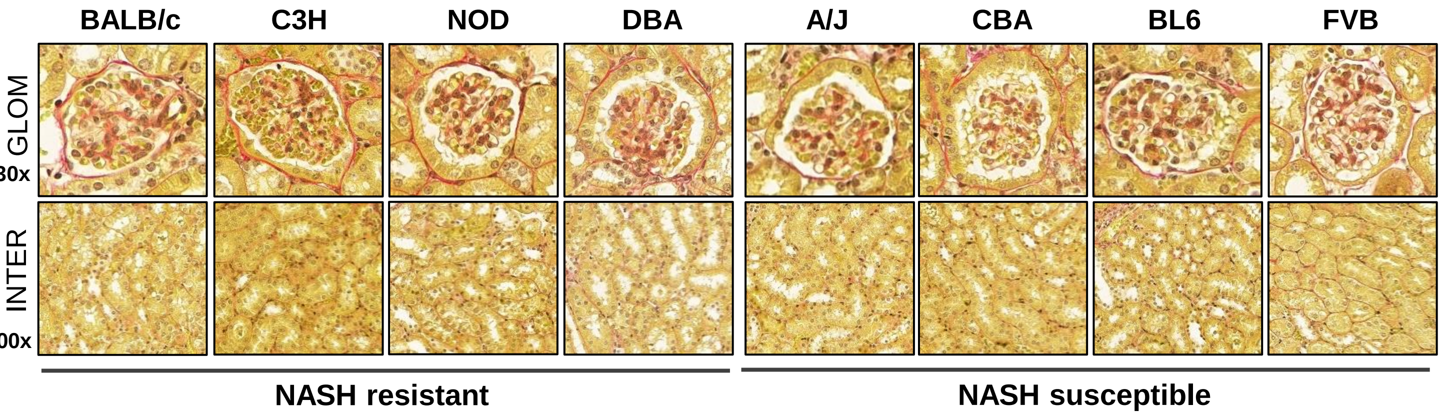

Figure S5. Kidney histopathology staining in all mouse strains fed a Control diet. Representative glomerular (GLOM) and interstitial (INTER) histology (Picrosirius Red staining) in A/J, BALB/c, C3H/HeJ, C57BL/6J, CBA/CaH, DBA/2J, FVB/N and NOD/ShiLtJ mice fed a Chow diet, sorted by increasing prevalence of NASH and liver fibrosis.

Figure S6

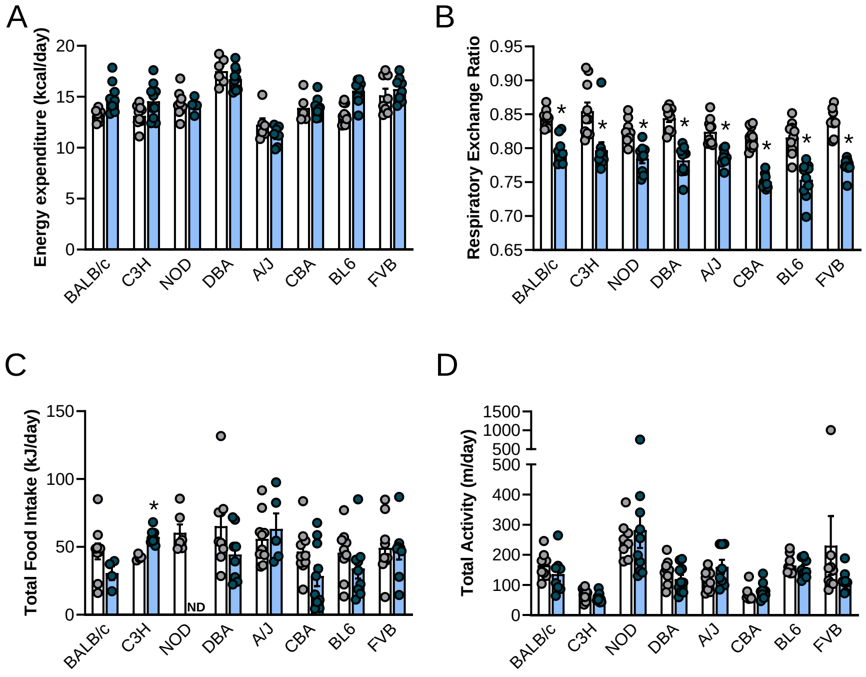

Figure S6. Assessment of systemic energy metabolism. Energy metabolism was assessed using Promethion Metabolic Cages, and data are shown for A/J, BALB/c, C3H/HeJ, C57BL/6J, CBA/CaH, DBA/2J, FVB/N and NOD/ShiLtJ mice fed the Chow and NASH diets, sorted by increasing prevalence of NASH and liver fibrosis. **(A)** Whole-body oxygen consumption (n=5-10/group), **(B)** respiratory exchange ratio (n=8-10/group), **(C)** food intake (n=4-10/group), and **(D)** locomotor activity (n=8-10/group). Data are means ± SEM, * $p<0.05$ vs. respective controls, as assessed by two-way unpaired t-test.

Karimkhanloo et al.

Mouse strain-dependent variation in metabolic associated fatty liver disease (MAFLD) – A comprehensive resource tool for pre-clinical studies

Supplementary Tables

Table S1. Composition of chow and NASH diet, as provided by Specialty Feeds (Glen Forrest, Western Australia, Australia)

	**Chow Diet (Specialty Feeds SF00-100)**	**NASH Diet (Specialty Feeds SF16-033)**
**Calculated Nutritional Parameters**		
Protein (%)	19.6	21.9
Total Fat (%)	4.4	20.0
Crude Fibre (%)	4.6	5.2
Acid Detergent Fibre (%)	7.70	5.20
Digestible Energy (MJ/kg)	14.0	18.4
Total Calculated Energy from Protein (%)	22.6	20.0
Total Calculated Energy from Lipids (%)	11.1	39.8
**Calculated Fatty Acids (%)**		
Myristic Acid (14:0)	0.02	0.26
Palmitic Acid (16:0)	0.52	4.85
Stearic Acid (18:0)	0.14	3.07
Palmitoleic Acid (16:1)	0.01	0.30
Oleic Acid (18:1)	1.81	6.46
Gadoleic Acid (20:1)	0.03	0.13
Linoleic Acid (18:2 n6)	1.41	3.90
a Linolenic Acid (18:3 n3)	0.29	0.43
Arachadonic Acid (20:4 n6)	Trace	Trace
EPA (20:5 n3)	0.02	no data
DHA (22:6 n3)	0.03	no data

Total n3	0.34	0.46
Total n6	1.42	3.92
Total Monounsaturated Fats	1.85	6.95
Total Polyunsaturated Fats	1.79	4.47
Total Saturated Fats	0.73	8.40
**Calculated Amino Acids (%)**		
Valine	0.87	1.39
Leucine	1.53	1.90
Isoleucine	0.92	1.05
Threonine	0.66	0.88
Methionine	0.26	0.92
Cysteine	0.34	0.08
Lysine	0.84	1.58
Phenylalanine	0.98	1.04
Tyrosine	0.70	1.10
Tryptophan	0.22	0.19
Histidine	0.53	0.58
Taurine (mg/kg)	83.4	no data
**Calculated Total Vitamins**		
Vitamin A (Retinol) (IU/kg)	11 080	4 450
Vitamin D (Cholecalciferol) (IU/kg)	2 000	1 110
Vitamin E (a Tocopherol acetate) (mg/kg)	110	85
Vitamin K (Menadione) (mg/kg)	20	1.1
Vitamin C (Ascorbic acid)	no data	none added
Vitamin B1 (Thiamine) (mg/kg)	84	6.8
Vitamin B2 (Riboflavin) (mg/kg)	30	7.0
Niacin (Nicotinic acid) (mg/kg)	145	34.0
Vitamin B6 (Pryridoxine) (mg/kg)	28	8.0
Pantothenic Acid (mg/kg)	60	18.4
Biotin (ug/kg)	410	222
Folic Acid (mg/kg)	5.0	2.3
Inositol	no data	none added

Vitamin B12 (Cyancobalamin) (ug/kg)	150	114
Choline (mg/kg)	1 630	1 780
**Calculated Total Minerals**		
Calcium (%)	0.82	0.66
Phosphorous (%)	0.70	0.44
Magnesium (%)	0.20	0.07
Sodium (%)	0.20	0.13
Potassium (%)	0.66	0.80
Sulphur (%)	0.17	0.24
Iron (mg/kg)	263	69
Copper (mg/kg)	24.0	9.0
Iodine (mg/kg)	0.5	0.2
Manganese (mg/kg)	113	20
Cobalt (mg/kg)	0.6	no data
Zinc (mg/kg)	94	53
Molybdenum (mg/kg)	1.3	0.17
Selenium (mg/kg)	0.4	0.3
Cadmium (mg/kg)	0.04	no data
Chromium (mg/kg)	no data	1.1
Fluoride (mg/kg)	no data	1.1
Lithium (mg/kg)	no data	0.1
Boron (mg/kg)	no data	2.0
Nickel (mg/kg)	no data	0.6
Vanadium (mg/kg)	no data	0.1

Table S2. Primer Sequences.

Primer	Forward	Reverse
Acta2	CATCTTTCATTGGGATGGAG	TTAGCATAGAGATCCTTCCTG
Adgre1	CCTGGACGAATCCTGTGAAG	GGTGGGACCACAGAGAGTTG
Ccl2	TATCAGAAGCAAAACGT	TCACGTTTGAATCAACTCAA
Ccn2 (Ctgf)	GAGGAAAACATTAAGAAGGGC	AGAAAGCTCAAACTTGACAG
Col1a1	CGTATCACCAAACTCAGAAG	GAAGCAAAGTTTCCTCCAAG
Hprt	AGGGATTTGAATCACGTTTG	TTTACTGGCAACATCAACAG
Tgfb1	GGATACCAACTATTGCTTCAG	TGTCCAGGCTCCAAATATAG
Timp1	CATCCTCTTGTTGCTA	CATGAATTTAGCCCTTATGACC
Tnf (TNFa)	CTGTTGAAGGAATGGGTGTT	GGTCACTGTCCCAGCATCTT

Table S3. Histopathological liver grading of Control and NASH mice, according to the Clinical Research Network NAFLD activity score (NAS) and Kleiner classification of liver fibrosis.

	Chow diet								NASH diet							
	**BALB**	**C3H**	**NOD**	**DBA**	**A/J**	**BL6**	**CBA**	**FVB**	**BALB**	**C3H**	**NOD**	**DBA**	**A/J**	**BL6**	**CBA**	**FVB**
**NAS**																
≤ 4	9	12	8	9	10	10	10	9	8	9	10	10	3	0	1	0
≥ 5	0	0	0	0	0	0	0	0	0	0	0	0	5	10	9	10
**Steatosis grade (%)**																
0 – ≤ 5%	9	12	8	7	9	10	9	9	8	3	3	3	1	0	0	0
1 – 5-33%	0	0	0	0	1	0	0	0	0	5	7	5	1	0	0	1
2 – 34-66%	0	0	0	2	0	0	0	0	0	1	0	2	3	1	0	2
3 – > 66%	0	0	0	0	0	0	1	0	0	0	0	0	3	9	10	7
**Lobular Inflammation**																
0 – none	9	12	8	9	8	10	10	9	8	9	6	7	1	0	1	0
1 – < 2	0	0	0	0	2	0	0	0	0	0	4	3	1	4	2	4
2 – 2-4	0	0	0	0	0	0	0	0	0	0	0	0	3	3	4	6
3 – > 4	0	0	0	0	0	0	0	0	0	0	0	0	3	3	3	0
**Ballooning**																
0 – none	7	11	6	3	4	4	4	6	5	4	3	3	1	0	1	0
1 – few	2	1	2	1	4	3	3	3	2	3	5	0	5	5	3	6
2 – many	0	0	0	5	2	3	3	0	1	2	2	7	2	5	6	4
**Fibrosis**																
0 – none	9	12	8	9	10	10	10	9	8	9	10	10	3	4	6	0
1a	0	0	0	0	0	0	0	0	0	0	0	0	3	4	4	8
1b	0	0	0	0	0	0	0	0	0	0	0	0	2	1	0	2
1c	0	0	0	0	0	0	0	0	0	0	0	0	0	1	0	0
2	0	0	0	0	0	0	0	0	0	0	0	0	0	0	0	0
3 – bridging	0	0	0	0	0	0	0	0	0	0	0	0	0	0	0	0

Table S4. Tissue weights and plasma profile in Control and NASH mice.

	Diet	BALB/c	C3H	NOD	DBA	A/J	BL6	CBA	FVB
**Fasting blood glucose (mmol/L)**	Chow	6.9 ± 0.2	7.6 ± 0.3	4.5 ± 0.2	7.1 ± 0.5	7.2 ± 0.2	7.2 ± 0.3	8.7 ± 0.2	7.9 ± 0.2
	NASH	7.1 ± 0.2	7.8 ± 0.3	5.7 ± 0.2*	7.6 ± 0.3	7.1 ± 0.1	8.1 ± 0.5	8.7 ± 0.2	8.5 ± 0.3
**total AUC (OGTT)**	Chow	959 ± 27	918 ± 35	820 ± 18	942 ± 62	1025 ± 40	1084 ± 33	1461 ± 102	1266 ± 35
	NASH	1013 ± 33	925 ± 27	888 ± 34	938 ± 28	1178 ± 61	1212 ± 47*	1222 ± 29*	1523 ± 69*
**Fasting plasma insulin (pmol/L)**	Chow	194 ± 30	299 ± 90	85 ± 15	1105 ± 357	182 ± 17	398 ± 102	933 ± 108	200 ± 20
	NASH	239 ± 15	620 ± 234	373 ± 71*	1644 ± 338	116 ± 29	421 ± 55	1338 ± 126*	505 ± 120*
**KITT (%/min)**	Chow	2.1 ± 0.3	1.9 ± 0.2	1.9 ± 0.2	1.3 ± 0.3	2.1 ± 0.3	1.8 ± 0.3	0.9 ± 0.2	2.4 ± 0.2
	NASH	1.8 ± 0.2	1.6 ± 0.3	1.9 ± 0.3	0.8 ± 0.3	1.8 ± 0.2	2.6 ± 0.4	1.0 ± 0.3	1.3 ± 0.3*
**HOMA-IR**	Chow	9.1 ± 2.2	25.2 ± 7.6	2.5 ± 0.4	58.6 ± 6.2	8.4 ± 1.3	17.7 ± 3.3	52.7 ± 6.1	10.2 ± 1.7
	NASH	11.4 ± 0.9	31 ± 12.7	16.5 ± 3.3*	75.1 ± 11.3	5.3 ± 1.0	22.2 ± 3.2	74.8 ± 1.9*	27.9 ± 8.1*
**Body weight (g)**	Chow	32 ± 0.9	37 ± 1.9	36 ± 0.8	37 ± 0.8	30 ± 1.2	36 ± 1.4	38 ± 1.0	35 ± 0.1
	NASH	38 ± 1.2*	41 ± 1.6	40 ± 1.8	46 ± 1.7*	31 ± 1.3	50 ± 1.3*	42 ± 0.1*	42 ± 1.2*
**Heart (mg)**	Chow	187 ± 8.7	159 ± 5.3	151 ± 12.2	212 ± 3.7	124 ± 4.2	153 ± 3.8	149 ± 3.8	167 ± 3.0
	NASH	184 ± 8.2	154 ± 9.9	133 ± 18.5	223 ± 5.5	108 ± 3.3*	164 ± 8.7	140 ± 8.4	164 ± 2.7
**Epididymal adipose tissue (g)**	Chow	1.0 ± 0.1	0.6 ± 0.1	0.5 ± 0.1	1.0 ± 0.1	1.0 ± 0.1	1.3 ± 0.2	1.1 ± 0.1	1.0 ± 0.1
	NASH	2.1 ± 0.2*	0.7 ± 0.1	1.7 ± 0.2*	1.4 ± 0.1*	1.0 ± 0.1	1.9 ± 0.1*	0.8 ± 0.04*	1.0 ± 0.1
**Inguinal adipose tissue (g)**	Chow	0.4 ± 0.1	0.4 ± 0.1	0.4 ± 0.02	1.0 ± 0.1	0.9 ± 0.1	0.6 ± 0.1	1.1 ± 0.1	0.6 ± 0.1
	NASH	0.8 ± 0.1*	1.0 ± 0.1*	0.8 ± 0.1*	2.0 ± 0.1*	1.0 ± 0.1	2.2 ± 0.2 *	1.4 ± 0.1	1.5 ± 0.1*
**Brown adipose tissue (mg)**	Chow	106 ± 7	77 ± 14	81 ± 6	269 ± 22	68 ± 5	87 ± 10	176 ± 15	163 ± 15
	NASH	127 ± 9	118 ± 17	121 ± 8*	432 ± 37*	58 ± 4	113 ± 13	208 ± 32	255 ± 27*
**Kidney (mg)**	Chow	284 ± 9	319 ± 12	270 ± 8	362 ± 14	170 ± 8	184 ± 20	308 ± 15	282 ± 13
	NASH	276 ± 13	311 ± 14	266 ± 9	310.1 ± 6*	164 ± 13	219 ± 8	275 ± 14	256 ± 7
**Quadriceps muscle (mg)**	Chow	450 ± 13	364 ± 15	387 ± 19	374 ± 10	302 ± 16	387 ± 18	385 ±14	414 ± 13
	NASH	436 ± 12	383 ± 18	377 ± 28	425 ±10*	287 ± 17	420 ±15	367 ± 12	434 ± 15
**Gastrocnemius muscle (mg)**	Chow	351 ± 15	284 ± 16	385 ± 25	280 ± 9	226 ± 11	329 ± 9	369 ± 18	373 ± 15
	NASH	340 ± 19	318 ± 14	342 ± 14	326 ± 9*	220 ± 11	337 ± 24	365 ± 15	356 ± 14

Shown are means ± SEM, n = 4-10 per group. * $p < 0.05$ as assessed by unpaired students t-test. AUC = total area-under-curve during the oral glucose tolerance test (OGTT). KITT = glucose disappearance rate for ITT (KITT; %/min) calculated as KITT=(0.693x100) /

t1/2, where t1/2 is the slope of the plasma glucose concentration during the first 20 min of the ITT. The HOMA-IR was calculated as: Fasting insulin (µU/L) x fasting glucose (nmol/L)/22.5
